# High-Quality Draft Genome Sequence of Desulfovibrio carbinoliphilus FW-101-2B, an Organic Acid-Oxidizing Sulfate-Reducing Bacterium Isolated from Uranium(VI)-Contaminated Groundwater

**DOI:** 10.1128/genomeA.00092-15

**Published:** 2015-03-12

**Authors:** Bradley D. Ramsay, Chiachi Hwang, Hannah L. Woo, Sue L. Carroll, Susan Lucas, James Han, Alla L. Lapidus, Jan-Fang Cheng, Lynne A. Goodwin, Samuel Pitluck, Lin Peters, Olga Chertkov, Brittany Held, John C. Detter, Cliff S. Han, Roxanne Tapia, Miriam L. Land, Loren J. Hauser, Nikos C. Kyrpides, Natalia N. Ivanova, Natalia Mikhailova, Ioanna Pagani, Tanja Woyke, Adam P. Arkin, Paramvir Dehal, Dylan Chivian, Craig S. Criddle, Weimin Wu, Romy Chakraborty, Terry C. Hazen, Matthew W. Fields

**Affiliations:** a Center for Biofilm Engineering, Montana State University, Bozeman, Montana, USA; b Department of Civil and Environmental Engineering, The University of Tennessee, Knoxville, Tennessee, USA; c Environmental Sciences Division, Oak Ridge National Laboratory, Oak Ridge, Tennessee, USA; d Department of Energy Joint Genome Institute, Walnut Creek, California, USA; e Physical Biosciences Division, Lawrence Berkeley National Laboratory, Berkeley, California, USA; f ENIGMA, Lawrence Berkeley National Laboratory, Berkeley, California, USA; g Department of Civil and Environmental Engineering, Stanford University, Stanford, California, USA; h Earth Sciences Division, Lawrence Berkeley National Laboratory, Berkeley, California, USA; i Department of Microbiology, The University of Tennessee, Knoxville, Tennessee, USA; j Department of Earth & Planetary Sciences, The University of Tennessee, Knoxville, Tennessee, USA; k Department of Microbiology & Immunology, Montana State University, Bozeman, Montana, USA

## Abstract

Desulfovibrio carbinoliphilus subsp. oakridgensis FW-101-2B is an anaerobic, organic acid/alcohol-oxidizing, sulfate-reducing δ-proteobacterium. FW-101-2B was isolated from contaminated groundwater at The Field Research Center at Oak Ridge National Lab after *in situ* stimulation for heavy metal-reducing conditions. The genome will help elucidate the metabolic potential of sulfate-reducing bacteria during uranium reduction.

## GENOME ANNOUNCEMENT

Desulfovibrio carbinoliphilus subsp. oakridgensis FW-101-2B was isolated from groundwater of well FW-101 at The Field Research Center (FRC) at Oak Ridge National Lab (ORNL). The FRC is part of the Y-12 security complex, located in the Bear Creek drainage. ICP-MS analysis of FW-101 groundwater in late 2001 estimated uranium concentrations between 20 and 250 ppm, and chromium concentrations between 35 and 85 ppm. Previous studies have demonstrated reduction of nitrate and uranium levels in the subsurface upon bio-stimulation with ethanol at the FRC (1–3). During bio-stimulation, an increase was observed in DNA sequences corresponding to sulfate-reducing bacteria. Groundwater from well FW-101 at the FRC site was collected during the uranium-reduction phase of a previously described biostimulation experiment (3) and used as inoculum for an enrichment culture for sulfate-reducing bacteria. The enrichment was grown at room temperature anaerobically in ES4D medium (pH 6.7). The ES4D medium has the same ingredients as a previously described medium, LS4D, except ethanol replaced the lactate (4).

The genome was sequenced by 454 GS FLX Titanium and paired-end Illumina GAii (2 × 35 bp). The pyrosequencing and Illumina reads were assembled using the Newbler (Roche) and Velvet (5), respectively. Phred/Phrap/Consed (http://www.phrap.com) was used for genome finishing. Genes were identified using Prodigal (6) and used to search the National Center for Biotechnology Information (NCBI) nonredundant database, UniProt, TIGRFam, Pfam, PRIAM, KEGG, COG, and InterPro databases. Additional annotation was performed within the (IMG-ER) platform (7).

Sequence determination revealed a 4.1-Mb genome with 66.5% G+C content, which is comparable to D. carbinoliphilus (63% G+C) and Desulfovibrio vulgaris (63.3% G+C). The COG predictions categorize 581 of the 3,737 protein-encoding genes as pertaining to information storage and processing, 1,183 as cellular processes, 1,576 as metabolism genes, and 596 as poorly characterized functions. Sequencing detected 2 plasmids of different sizes and G+C content, pFW10101 (97,864 bp, 67% G+C) and pFW10102 (21,111 bp, 57.5% G+C).

FW-101-2B was most closely related to D. carbinoliphilus D41^T^. The ANIb values calculated by JSpecies (8) showed that FW-101-2B was more similar to D. vulgaris Miyazaki (69.11%), D. vulgaris Hildenborough (67.45%), and D. vulgaris DP4 (67.41%) than Syntrophobacter fumaroxidans (64.68) and Desulfovibrio desulfuricans ATCC 27774 (60.93%). The small-subunit (SSU) rRNA gene and sulfite reductase gene (*dsrAB*) of FW-101-2B was most similar to D. carbinoliphilus D41^T^ at 99% and 92% similarity, respectively.

Although FW-101-2B is phylogenetically very similar to D. carbinoliphilus D41^T^, physiological evidence would support classification of a new strain. Thus, we propose the classification as D. carbinoliphilus subsp. oakridgensis. This is the first genome sequence of a D. carbinoliphilus strain.

### Nucleotide sequence accession numbers.

The whole-genome shotgun project has been deposited at DDBJ/EMBL/GenBank under the accession no. ADFE00000000. The version described in this paper is the version ADFE00000000.2.
